# Types of colourants used in tattoo and permanent make‐up techniques, legal regulations, health, and psychological aspects of tattooing

**DOI:** 10.1002/hsr2.1360

**Published:** 2023-09-08

**Authors:** Anna Charuta, Robert Wegner, Kamila M. Charuta, Karolina Hanusek, Agnieszka Paziewska

**Affiliations:** ^1^ Institute of Health, Faculty of Medical and Health Sciences Siedlce University of Natural Sciences and Humanities Siedlce Poland; ^2^ SWPS University of Social Sciences and Humanities Poland; ^3^ Centre of Postgraduate Medical Education Warsaw Poland

**Keywords:** black ink for tattoos, coloured ink for tattoos, inflammatory reactions, legal acts regulating the use of ink for tattoos, pigments, REACH

## Abstract

**Background:**

It is estimated that more than 60 million people in Europe, that is, around 12% of the European population, have at least one tattoo. However, there is still little information on the long‐term effects of tattoos. Inks used for tattooing are a mixture of chemicals, with pigments being the main components responsible for the visual effect. The pigments used are not produced specifically as ingredients for tattooing, but mainly/primarily for the needs of industry, where lower purity requirements and quality standards are acceptable. It is therefore necessary to understand the risks associated with tattoos, but also to implement appropriate legal regulations. The aim of this article was to collect and summarise the results of research conducted so far on the type of colourants used in tattoo ink and to analyze the impact of these on human health. In addition, as part of this work, the current legal acts regulating the concentration limits and composition of inks used in tattooing as well as the psychological aspects of tattooing were collected and presented.

**Methods:**

Scientific reports and articles from renowned journals from 1994 to 2022, relevant review and research publications in PubMed, and Google Scholar were analyzed. To analyze the available research literature, the Web of Science, Scopus, PubMed databases were used. The following keywords were used to search for publications: tattoos, colourants used in tattoos, side effects of tattoos, legal acts, psychological aspects of tattoos.

**Results:**

The result of the literature analysis indicates a risk to health and side effects associated with tattooing the body. There are still no standardised test methods to analyze tattoo inks and assess their safety. Although the art of tattooing has been known for millennia, European legal authorities have not yet implemented effective regulations. Currently, tattoo products in Europe are covered by the general REACH regulation (Resolution ResAP, 2008; EU regulation 2020/2081, 2020). on product safety. The new amendment in force since January 4, 2022 introduces concentration limits for certain substances used in tattoo and permanent makeup inks. However, these provisions do not sufficiently protect either the consumer or the tattoo industry.

**Conclusions:**

The results of the research indicate a potentially harmful effect on skin health. A more stringent safety assessment of the colourants used for tattooing is recommended, supported by studies and applicable legislation.

## 1 INTRODUCTION

The tattoo is a permanent mark or pattern on the skin, a modification of the body made by introducing a colourant under the skin. The word tattoo comes from the Tahitian language, where the word “tatau” means “to mark something,” and the word itself originates from the extension of the core “ta” meaning “to tap or hit”.

It is difficult to determine exactly when the art of tattooing was created. According to historical sources and archeological evidence (preserved mummified skin), tattooing has been practiced all over the world at least since the Neolithic period. Tools found which were probably used for tattooing even point to the period of the Upper Palaeolithic as the beginning of this practice in Europe, in areas of France, Portugal and Scandinavia. Direct evidence of tattooing is presented by the tattooed mummified human skin of an ancient Alpine mummy called Ötzi, dated 3370−3100 BC.

Currently, it is estimated that more than 60 million people in Europe, that is, around 12% of the European population, have at least one tattoo. Moreover, the number of people with tattoos or permanent make‐up in the European Union is steadily increasing, especially among young people. These can be tattoos used to decorate the body permanently through the invasive introduction of pigments into the dermis (to a depth of approx. 1−2 mm) using a tattoo machine or in an impermanent, temporary manner made with a natural substance on the surface of the skin. A painless, decorative, and above all temporary tattoo (lasting up to 3 weeks) can be obtained thanks to the application of henna, plant extract from the plant *Lawsonia inermis*.^1^ The process of permanent tattooing involves the introduction of pigments into the skin by puncture so as to obtain a durable, specific pattern. The injected dye is identified by the immune system as a foreign substance, is absorbed by monocytes, and is encapsulated in fibroblasts in the dermis.

A prototype of tattoo ink first appeared in 1950, developed by Milton Zeis. Lead‐based pigments were sold in powder form and required mixing with distilled water. Knowledge of the toxicity of ink components and the dangers associated with it was negligible. Mixtures of pigments aimed at obtaining different colors were characterized by a complex composition, usually not disclosed by manufacturers.

## 2 TATTOO INK—INGREDIENTS, IMPURITIES

During the procedure of tattooing into the skin using rotating needles tattoo machines, a large amount of ink is injected, about 0.4−14.36 mg of pigment per 1 cm^2^ skin. These mixtures are introduced into the human dermis, but also into the inside of the eyeball or mucous membranes. The inks used for tattooing contain a multi‐component mixture of chemical compounds, with the colouring components (pigments and colourants) being the main components responsible for the visual effect and color. Dyes are suspended in a solvent, most often in water or alcohol and supplemented with other auxiliary components, such as preservatives and thickeners or binders.^2^, ^3^, ^4^, ^5^, ^6^ The pigments used (organic colourants which are dominant in inks, inorganic colourants and metal salts of different colors) are often not produced specifically for tattooing, but rather for industrial applications such as paints (including azo dyes), coatings, plastics (aromatic amines) or textiles, where lower purity requirements are allowed.^3^ In addition, no information about their purity, quality or composition is required. Furthermore, it is common practice for tattoo artists to prepare their own mixtures to obtain a better visual effect or color, which makes it very difficult to determine the content of substances present in the applied ink, including these harmful substances.^3^, ^4^, ^5^

Among the components that are found in the inks are impurities. The inks contain heavy metals such as chromium (green and blue colourants), cobalt (e.g., yellow and blue colourants), lead, antimony, arsenic, beryllium as well as nickel and mercury (red colourant) often classified by the IARC as carcinogenic or potentially carcinogenic compounds. In addition, the presence of aromatic amines, phthalates, polycyclic aromatic hydrocarbons, and nanoparticles has been confirmed. However, these ingredients are not included on ink labels and their presence in inks is even prohibited in Europe (cadmium, lead, chromium, and copper).

Although tattoo colourants are injected subcutaneously, their suspensions are not classified as medical or pharmaceutical substances. They also do not have to meet the certification standards of products intended for injection and their ingredients are often not properly tested.^7^ Oftentimes, the quality of the ink depends on the standards applied by a particular manufacturer. The components of tattoo ink are frequently undefined and also highly variable. They may include allergenic compounds, or be potentially toxic or carcinogenic.

## 3 TATTOOING—THE HEALTH ASPECT

The procedures used in applying a tattoo or permanent makeup (together understood as tattooing), regardless of whether they involve the use of needles or some other technique such as microblading, result in a disruption of the continuity of the tissue, damage to the skin barrier, epidermis, and dermis.

The depth of injection/application and the amount of ink depends on the type and properties of the mixture (e.g., density), components, equipment, skills of the tattoo artist, and type of tattoo.

The chemical structure of pigments, the size of molecules, their insolubility, and hydrophobic properties mean that little is known about the distribution of ink particles throughout the body and organs after tattooing. Damage to the epidermis and skin causes an immediate inflammatory reaction (by recruiting macrophages, neutrophils, T lymphocytes, among others) and pain, swelling or bleeding. Colourants usually accumulate close to the place where the mixture was applied, so that the tattoo or permanent makeup remains visible. The ink components are absorbed by macrophages and fibroblasts. The soluble components of the mixture are distributed within a few hours or days throughout the body and their concentration in the skin decreases over a period of several weeks.

Engel et al.^8^ proved that part of the pigment remains at the injection site, and its remaining nanoparticles enter the lymph nodes via the bloodstream, and it is possible that they are transported and accumulated in larger organs of the body along with impurities which may be present.

The migration of nanoparticle‐sized compounds in the body was confirmed by scientists from China (Tang et al.^9^) by subcutaneously injecting nanoparticles and silver microparticles into experimental rats. Microparticles have been found to be unable to enter the bloodstream, unlike nanoparticles, which have been transported to the kidneys, liver, spleen, lungs, and brain.^8^, ^9^

The components of the ink can therefore be deposited in the lymph nodes, other organs (such as the liver) or be excreted in the faeces or with urine. The metabolism of ink components and the method of their excretion are still not fully understood due to the small amount of scientific research conducted.

However, in recent years, there have been more and more clinical and scientific reports of direct and indirect health effects that can be caused by the application of tattoos.

The most common of these are acute or chronic skin reactions; allergic or inflammatory infections are often associated with both bacterial infections (including *Staphylococcus aureus*, *Streptococcus pyogenes*, Pseudomonas aeuroginosa, Clostridium dificile, or others), viruses (HPV, HCV, HBV MCV, HSV, and HIV), or more rarely fungal or parasitic infections. They manifest themselves as eczema reactions, warts, ulcers, dermatoses (lichens, granulomas), and hypersensitivity reactions (PMID: 34969030). The reason for these reactions are irregularities both during the tattooing procedure itself (contaminated tools, lack of skin disinfection, insufficient occupational and workplace hygiene) and those resulting from improper care of tattooed skin and failure to observe hygienic recommendations and disinfection by the tattooed person.

Allergic reactions caused by ink components and impurities may manifest themselves as plaque infiltrates, peeling, ulcers, eczema, erythema, papular or vesicular lesions, contact dermatitis (henna mixture with paraphenylenediamine PPD), sarcoidosis, and itching, burning or pain (PMID: 34605159).^9^, ^10^ The greatest allergenic potential is shown by inks containing mercury salts (red, pink, and orange), and slightly less often by inks whose pigment contains cobalt salts (blue, green, and yellow). Allergic reactions may occur during or after wound healing. Especially in the case of late allergic reactions that occur after weeks, months or years, it is difficult to identify the specific component or components of the ink that trigger the reaction. In the last decade, there has also been a rapid development of semi‐permanent tattoos also known as micropigmentation, where colourants are introduced into the second or third layer of the epidermis (approx. 0.3−0.5 mm) and serve mainly to improve the appearance of eyebrows, eyelids, and lip contour.^11^ A widely used group of pigments in permanent makeup are inorganic pigments which entail a lower risk of allergic reactions. This may be related to the size of the colourant molecule and the shallower application of the tattoo in the skin.^12^

Some components of tattoo inks can be dangerous and sensitizing chemicals of unknown potential. Until now, these allergens have been identified only in rare cases. Methods and experimental approaches for identifying allergens in tattoo inks through patient testing and in vitro methods have been described by Weiß KT.^13^

Currently, research aimed at analyzing the effect of tattooing on the functioning of the body is also being carried out on reconstructed human skin. Bil W, 2018 in his research tested two red and three black inks (Intenze (Intenze Products, Kalsdorf, Austria), Eternal Ink (Eternal Ink) and Carbon Black (H‐A‐N) for tattooing on a reconstructed epidermis on a collagen hydrogel filled with fibroblasts (3D organotype RHS model).^14^ He showed that all but one of the inks displayed cytotoxicity, while two of them (Eternal Ink Light Red and Intenze Sculpting Black) were able to induce an inflammatory reaction (IL‐18) and, consequently, allergic contact dermatitis after exposure to these tattoo inks.

Colourant metabolism in the skin and exposure of tattooed skin to sunlight or laser light can lead to the breakdown of pigment molecules, the formation of new and potentially toxic or carcinogenic compounds, and their release from the tattooed area of the body. The breakdown of pigment into smaller molecules allows them to be further distributed throughout the body (with the participation of macrophages) and accumulated in distant organs, mainly lymph nodes, but the exact fate of such molecules is not sufficiently understood. The presence of a tattoo is also associated with intolerance to sunlight, as pigments absorb UV radiation, degrade and induce processes leading to the formation of reactive oxygen species responsible for cell damage.

The results of scientific research on the influence of tattoo ink components on the initiation of carcinogenesis remain inconclusive. Despite the fact that some of the substances found in tattoo inks are carcinogenic (including cadmium, chromium, aromatic hydrocarbons, o‐toluidine and others), the direct relationship between tattooing and cancer induction has not yet been determined. One review (from 2012) concluded that any links between skin cancers arising in tattoos “should so far be considered coincidental.” Another review from 2016 maintains that adverse reactions are “relatively rare” and “generally unpredictable” and are mainly related to the immune system or skin infections. In a paper published in Current Oncology in 2021, cases of melanoma, basal cell carcinoma, squamous cell carcinoid, or squamous cell skin cancer were described which developed at the tattoo site. These melanomas or basal cell carcinomas were more common in the case of dark colourants such as black or navy blue, while squamous cell carcinomas and carcinoids developed after exposure of the skin to red colourants.

## 4 LEGAL REGULATIONS CONCERNING SUBSTANCES INCLUDED IN TATTOO INK

Both tattoo inks and tattooing have not been covered by sufficiently precise consumer safety rules for a long time.^15^ There is also a lack of regulation of the tattoo profession. In addition, no permissions are required to make tattoos. Amateur tattoo parlours, along with the risk of noncompliance with sanitary rules and unlicensed, insufficiently controlled tattoo products, pose a serious risk to the health of the tattooed person. Many of the chemicals currently used in inks have not yet been approved by the European Chemicals Agency (ECHA) or the Food and Drug Administration. They are also not recognised as medical products, despite the fact that they are applied under the skin, which requires breaking the continuity of the skin. Currently, therefore, individual countries are gradually taking steps to improve the safety of tattoo ink.

The first safety regulations were developed and approved by the Council of Europe as resolutions ResAP (https://rm.coe.int/16805df8e5) 2 and ResAP (https://rm.coe.int/16805d3dc4). The most important requirement of these resolutions is that inks do not endanger human health or safety. In the European Union, the use of many chemical substances (including aromatic amines) contained in inks has been restricted. Limits of concentrations of heavy metals, polycyclic aromatic hydrocarbons (PAH), and harmful substances considered to be mutagenic or carcinogenic have been defined (Council of Europe, https://eur-lex.europa.eu/legal-content/EN/TXT/?uri=celex%3A32008R1272).

In a subsequent EU Commission Regulation of December 14, 2020 concentration limits for carcinogenic, mutagenic, sensitising, corrosive and irritating components were introduced and a list of substances with specific concentration limits (including mercury lead, nickel, cadmium, arsenic, bar cadmium, cobalt, copper, zinc, lead, selenium, benzopyrene, and aromatic amines) was provided. In addition, the regulation stipulates the need to mark the ink labels with the composition contained.

To unify the rules, on January 4, 2022, the European Union introduced a new REACH (Regulation, Authorization and Restriction of Chemicals) regulation, which sets the most stringent requirements for tattoo inks to ensure the safety of their use for health, the environment and at the same time increase the competitiveness of the chemical industry in the EU. REACH completely replaces Resolution ResAP (https://rm.coe.int/16805d3dc4).^3^, ^12^, ^16^ The use of about 4000 dangerous chemical compounds in tattoo and permanent makeup inks that cause cancer, genetic mutations or are toxic to reproduction, are allergenic, corrosive or irritating to the skin and eyes has been restricted. According to the regulation, manufacturers and thus tattoo parlours are obliged to provide information about the composition of the ink they use to make the tattoo (labels on the packaging with information about the composition). To protect European citizens, dangerous substances in tattoo and permanent makeup inks are subject to restrictions on use in the EU under REACH. In addition, the regulations forbid the use of 44 prohibited pigments.

The regulations introduced to protect health are associated with serious consequences and difficulties for the entire tattoo industry. Restrictions on the use of components in tattoo mixtures (as well as binding agents and preservatives) and strict concentration limits on many substances contained in tattoo inks significantly restrict their use.

The industry's response to REACH is petition 1072/2020. The restrictions resulting from the regulation raise the concerns of the authors of the petitions regarding negative economic effects for the tattoo industry, but also concerning potentially adverse health effects. They point to the fact that the requirements that tattoo parlours would have to meet would force them to significantly increase prices, and limit the available products and ink colors. The authors of the petition expressed concern that some consumers, unable to obtain a tattoo in a reputable salon that complies with the regulations and applies legally allowed inks, will use the services of noncertified salons using inks not approved by the EU. In addition, it is possible that customers will use the possibility of traveling outside the EU to achieve the desired type or color of the tattoo. This may lead to the development of a market for illegal parlours using noncertified inks with lower sanitary standards.

There is also a shortage of substitutes in the market that would meet EU standards. Tattoo artists have made an attempt to develop new inks or new ink components. However, it remains an open question whether they will provide a similar intensity of color and durability of the tattoo as the substances used today. It is also problematic to produce or find substitutes for two specific pigments in blue and green, “Blue 15” and “Green 7,” before the deadline of January 2023. This raises concerns about the distant economic effects also affecting part of the cosmetic industry (permanent makeup) and medical tattoos (reconstructive surgery, including nipple reconstruction).

## 5 PSYCHOLOGICAL ASPECTS OF TATTOOING

Tattooing is one of the oldest forms of decorating the body. The art of tattooing has roots that go back far into the past, into tribal and pagan traditions associated with ritual or religious rites. It is not only a colourful drawing or image made on the body, but often also a symbol, a way of giving the body its own personal characteristics.

The available literature indicates specific personality traits of people who have undergone tattooing. The tattooing process involves people with a greater tendency to take any kind of risky action, often illegal or even to engage in criminal behavior. There is also a high correlation between the presence of tattoos and the use of numerous drugs, e.g. smoking, alcohol and marijuana abuse.^15^, ^16^, ^17^, ^18^, ^19^, ^20^ People with a tendency to tattoo their own body exhibited antisocial behaviors, showed a tendency to risky sexual behaviors,^21^, ^22^ deviance ^23^ or even suicide attempts.^24^

The tattoo is a kind of message about social status, personality, and health.^25^ Some researchers argue that tattoos may be a visible sign of “good genes.”^26^, ^27^ Koziel et al.^26^ showed in their research that tattooed men were more symmetrical, which may reflect a greater ability to overcome the challenges posed by the environment. Research by Hawkes suggests that tattoos give a sense of power and self‐control. Tattoos are also given a deeper psychological meaning.^27^ Body modifications may have enabled people to cope with unpleasant experiences after traumatic events.^28^, ^29^ Atkinson showed that tattooing has a therapeutic effect among women who have been abused.^28^ During the painful process of tattooing, these women re‐create representations of the wronged worship of their body, thanks to which they feel that they “recover their body”.

Armstrong, McConnell in 1994 distinguished three motives for choosing a tattoo: aesthetic, individual, and social. The aesthetic motif expresses the idea “I like it,” the individual motif in turn says, “I would like to express myself,” while the social motif is motivated by a desire to say, “I would like to show belonging to a group.”^16^ Another, more extensive classification was proposed by Wohlrab et al.^30^ It distinguishes the following types of motivation for tattooing: Table, which is still consistent with the emerging reports.

**Table:** 

1.	beauty art and fashion	1. tattoo as a fashion accessory or work of art^31^
2.	individuality	1. one of the most important motivations 2. having control over one's own body 3. creating one's own identity^32^
3.	personal narrative	1. expressing personal values and experiences through tattooing^33^
4.	physical endurance	1. a declaration on verifying one's resistance to pain and overcoming one's own limitations 2. courage^28^ 3. manifestation of auto‐aggressive tendencies^34^
5.	group affiliations and commitment	1. belonging to a specific community or subculture^35^ 2. involvement in the activities of a given group^34^
6.	resistance	1. protest against parents or society 2. protest during adolescence against parents^36^
7	spirituality and cultural tradition	1. personal tattoo 2. emphasizes belonging to specific cultures and their spiritual dimension^37^
8.	addiction	1. addictive nature of tattoos (endorphins released under the influence of the painful penetration of the needle or during tattooing) 2. anaesthesia during tattooing triggers positive sensations^38^
9.	sexual motivation	1. accentuation of one's sexuality 2. highlighting gender preference^39^, ^40^, ^41^
10.	no specific reason	1. getting the tattoo was an impulse decision^42^ 2. under the influence of alcohol or drugs 3. no reason or forgetting the reason for the tattoo^43^

Over recent years, however, tattoos have changed significantly in importance. Early on, Craik^44^ and Turner^45^ observed that tattoos are often perceived only as a fashion accessory. To take greater care of their external image, people underwent treatments aimed at improving their appearance, including tattooing the body.^46^

Currently, tattooing is a practice significantly rooted in modern society and is mainly of an aesthetic nature. The growing interest in tattooing is, among other things, the result of fashion among celebrities publishing images of their bodies decorated with tattoos. In addition, the greater availability and development of tattooing techniques that allow for the creation of very realistic, colourful and spatial images has changed the nature of the tattoo, the target group, and the social aspect. Currently, people very consciously decide to have precise, large and colourful tattoos done. They accept the need to allocate both their time and significant financial resources for this purpose. The assumption of the tattoo is to express, first of all, the personality and value of the tattooed person. The most common and basic motif of tattooing is self‐expression, a sense of identity and uniqueness, generating or strengthening one's own individuality or individual symbols indicating belonging to a specific group. The tattoo often commemorates a personal event, a stage in life or a close person or even an animal. In addition, it gives a sense of security or closeness to the person depicted on the tattoo. S. Weiler, 2021 based on the NfU‐G test (The Need for Uniqueness in German speaking populations, which determines the degree to which an individual is motivated to look unique and differ from others) showed that people with tattoos, piercings and other body modifications had higher needs for a sense of uniqueness than people without body modifications (PMID: 33657106).^47^ Moreover, people with tattoos took into account the social aspect, not being afraid of other people's reactions to their tattoos, although at the same time they did not want to evoke negative emotions. Tattoos are often acquired by people who are motivated to look special and stand out from others. (PMID: 33123650). The higher need to express uniqueness according to the opinion of Wahirab gives rise to the need for a tattoo. Then the tattoo is an expression of identity and a symbol of one's own individuality and uniqueness. Its presence, pattern or form may be associated with being or feelings, although it may also be a sign of anxiety or malaise.

## 6 SUMMARY

Clinical observations and available scientific literature indicate that tattoo inks may have a detrimental effect on skin health, body function, and cause delayed complications.

Despite the likely adverse economic consequences for the tattoo industry, the knowledge of the possible negative side effects on health does not allow us to remain passive. Extensive research is therefore needed on the health effects of the substances in inks and on the risk assessment of indirect allergic reactions and potentially carcinogenic effects. A much stricter safety assessment of tattoo inks and their components will enable further additional legal regulations to be developed, which will also lay down clear rules for the tattoo industry.

## AUTHOR CONTRIBUTIONS

**Anna Charuta**: Conceptualization; data curation; formal analysis; funding acquisition; investigation; methodology; project administration; supervision; writing—original draft. **Robert Wegner**: Investigation; writing—original draft. **Kamila M. Charuta**: Conceptualization; data curation; formal analysis; writing—original draft. **Karolina Hanusek**: Data curation; formal analysis; writing—original draft. **Agnieszka Paziewska**: Conceptualization; data curation; formal analysis; writing—original draft.

## CONFLICT OF INTEREST STATEMENT

The authors declare no conflict of interest.

## TRANSPARENCY STATEMENT

The lead author Anna Charuta affirms that this manuscript is an honest, accurate, and transparent account of the study being reported; that no important aspects of the study have been omitted; and that any discrepancies from the study as planned (and, if relevant, registered) have been explained.

## Supporting information

Supporting information.

## Data Availability

Data available on request from the authors
