# Supplementary material for: Types of colourants used in tattoo and permanent make‐up techniques, legal regulations, health, and psychological aspects of tattooing
Source: Health Sci Rep. 2023 Sep 8;6(9):e1360. doi: 10.1002/hsr2.1360 (PMC10485912; doi:10.1002/hsr2.1360)
Supplement: Supplementary file 1 — Supporting information. [file HSR2-6-e1360-s001.docx]

Supplement S1. **COMMISSION REGULATION (EU) 2020/2081 of 14 December 2020 amending Annex XVII to Regulation (EC) No 1907/2006 of the European Parliament and of the Council concerning the Registration, Evaluation, Authorisation and Restriction of Chemicals (REACH) as regards substances in tattoo inks or permanent make-up**

Substances falling within one or more of the following points:

1. substances classified as any of the following in Part 3 of Annex VI to Regulation (EC) No 1272/2008: — carcinogen category 1A, 1B or 2, or germ cell mutagen category 1A, 1B or 2, but excluding any such substances classified due to effects only following exposure by inhalation — reproductive toxicant category 1A, 1B or 2 but excluding any such substances classified due to effects only following exposure by inhalation — skin sensitiser category 1, 1A or 1B — skin corrosive category 1, 1A, 1B or 1C or skin irritant category 2 — serious eye damage category 1 or eye irritant category 2
2. substances listed in Annex II to Regulation (EC) No 1223/2009 of the European Parliament and of the Council (*)
3. substances listed in Annex IV to Regulation (EC) No 1223/2009 for which a condition is specified in at least one of the columns g, h and i of the table in that Annex
4. substances listed in Appendix 13 to this Annex. The ancillary requirements in paragraphs 7 and 8 of column 2 of this entry apply to all mixtures for use for tattooing purposes, whether or not they contain a substance falling within points (a) to (d) of this column of this entry.
5. **Shall not be placed on the market in mixtures for use for tattooing purposes, and mixtures containing any such substances shall not be used for tattooing purposes, after 4 January 2022 if the substance or substances in question is or are present in the following circumstances:**
6. in the case of a substance classified in Part 3 of Annex VI to Regulation (EC) No 1272/2008 as carcinogen category 1A, 1B or 2, or germ cell mutagen category 1A, 1B or 2, the substance is present in the mixture in a concentration equal to or greater than 0,00005 % by weight;
7. (b) in the case of a substance classified in Part 3 of Annex VI to Regulation (EC) No 1272/2008 as reproductive toxicant category 1A, 1B or 2, the substance is present in the mixture in a concentration equal to or greater than 0,001 % by weight;
8. in the case of a substance classified in Part 3 of Annex VI to Regulation (EC) No 1272/2008 as skin sensitiser category 1, 1A or 1B, the substance is present in the mixture in a concentration equal to or greater than 0,001 % by weight;
9. in the case of a substance classified in Part 3 of Annex VI to Regulation (EC) No 1272/2008 as skin corrosive category 1, 1A, 1B or 1C or skin irritant category 2, or as serious eye damage category 1 or eye irritant category 2, the substance is present in the mixture in a concentration equal to or greater than: (i) 0,1 % by weight, if the substance is used solely as a pH regulator; (ii) 0,01 % by weight, in all other cases;
10. in the case of a substance listed in Annex II to Regulation (EC) No 1223/2009 (*), the substance is present in the mixture in a concentration equal to or greater than 0,00005 % by weight;
11. in the case of a substance for which a condition of one or more of the following kinds is specified in column g (Product type, Body parts) of the table in Annex IV to Regulation (EC) No 1223/2009, the substance is present in the mixture in a concentration equal to or greater than 0,00005 % by weight: (i) “Rinse-off products”; (ii) “Not to be used in products applied on mucous membranes”; (iii) “Not to be used in eye products”;
12. in the case of a substance for which a condition is specified in column h (Maximum concentration in ready for use preparation) or column i (Other) of the table in Annex IV to Regulation (EC) No 1223/2009, the substance is present in the mixture in a concentration, or in some other way, that does not accord with the condition specified in that column;
13. in the case of a substance listed in Appendix 13 to this Annex, the substance is present in the mixture in a concentration equal to or greater than the concentration limit specified for that substance in that Appendix.
14. For the purposes of this entry use of a mixture “for tattooing purposes” means injection or introduction of the mixture into a person’s skin, mucous membrane or eyeball, by any process or procedure (including procedures commonly referred to as micro-pigmentation), with the aim of making a mark or design on his or her body.

3. If a substance not listed in Appendix 13 falls within more than one of points (a) to (g) of paragraph 1, the strictest concentration limit laid down in the points in question shall apply to that substance. If a substance listed in Appendix 13 also falls within one or more of points (a) to (g) of paragraph 1, the concentration limit laid down in point (h) of paragraph 1 shall apply to that substance.

4. By way of derogation, paragraph 1 shall not apply to the following substances until 4 January 2023: (a) Pigment Blue 15:3 (CI 74160, EC No 205-685-1, CAS No 147-14-8); (b) Pigment Green 7 (CI 74260, EC No 215-524-7, CAS No 1328-53-6).

5. If Part 3 of Annex VI to Regulation (EC) No 1272/2008 is amended after 4 January 2021 to classify or re-classify a substance such that the substance then becomes caught by point (a), (b), (c) or (d) of paragraph 1 of this entry, or such that it then falls within a different one of those points from the one within which it fell previously, and the date of application of that new or revised classification is after the date referred to in paragraph 1 or, as the case may be, paragraph 4 of this entry, that amendment shall, for the purposes of applying this entry to that substance, be treated as taking effect on the date of application of that new or revised classification.

6. If Annex II or Annex IV to Regulation (EC) No 1223/2009 is amended after 4 January 2021 to list or change the listing of a substance such that the substance then becomes caught by point (e), (f) or (g) of paragraph 1 of this entry, or such that it then falls within a different one of those points from the one within which it fell previously, and the amendment takes effect after the date referred to in paragraph 1 or, as the case may be, paragraph 4 of this entry, that amendment shall, for the purposes of applying this entry to that substance, be treated as taking effect from the date falling 18 months after entry into force of the act by which that amendment was made.

7. Suppliers placing a mixture on the market for use for tattooing purposes shall ensure that, after 4 January 2022, the mixture is marked with the following information:

(a) the statement “Mixture for use in tattoos or permanent make-up”;

(b) a reference number to uniquely identify the batch;

(c) the list of ingredients in accordance with the nomenclature established in the glossary of common ingredient names pursuant to Article 33 of Regulation (EC) No 1223/2009, or in the absence of a common ingredient name, the IUPAC name. In the absence of a common ingredient name or IUPAC name, the CAS and EC number. Ingredients shall be listed in descending order by weight or volume of the ingredients at the time of formulation. “Ingredient” means any substance added during the process of formulation and present in the mixture for use for tattooing purposes. Impurities shall not be regarded as ingredients. If the name of a substance, used as ingredient within the meaning of this entry, is already required to be stated on the label in accordance with Regulation (EC) No 1272/2008, that ingredient does not need to be marked in accordance with this Regulation; ENOfficial Journal of the European Union 15.12.2020 L 423/13

(d) the additional statement “pH regulator” for substances falling under point (d)(i) of paragraph 1;

(e) the statement "Contains nickel. Can cause allergic reactions." if the mixture contains nickel below the concentration limit specified in Appendix 13;

(f) the statement "Contains chromium (VI). Can cause allergic reactions." if the mixture contains chromium (VI) below the concentration limit specified in Appendix 13;

(g) safety instructions for use insofar as they are not already required to be stated on the label by Regulation (EC) No 1272/2008. The information shall be clearly visible, easily legible and marked in a way that is indelible. The information shall be written in the official language(s) of the Member State(s) where the mixture is placed on the market, unless the Member State(s) concerned provide(s) otherwise. Where necessary because of the size of the package, the information listed in the first subparagraph, except for point (a), shall be included instead in the instructions for use. Before using a mixture for tattooing purposes, the person using the mixture shall provide the person undergoing the procedure with the information marked on the package or included in the instructions for use pursuant to this paragraph.

8. Mixtures that do not contain the statement “Mixture for use in tattoos or permanent make-up” shall not be used for tattooing purposes.

9. This entry does not apply to substances that are gases at temperature of 20 °C and pressure of 101,3 kPa, or generate a vapour pressure of more than 300 kPa at temperature of 50 °C, with the exception of formaldehyde (CAS No 50-00-0, EC No 200-001-8).

10. This entry does not apply to the placing on the market of a mixture for use for tattooing purposes, or to the use of a mixture for tattooing purposes, when placed on the market exclusively as a medical device or an accessory to a medical device, within the meaning of Regulation (EU) 2017/745, or when used exclusively as a medical device or an accessory to a medical device, within the same meaning. Where the placing on the market or use may not be exclusively as a medical device or an accessory to a medical device, the requirements of Regulation (EU) 2017/745 and of this Regulation shall apply cumulatively.
